# An Improved Method for Deriving the Heat Source Model for FCAW of 9% Nickel Steel for Cryogenic Tanks

**DOI:** 10.3390/ma16206647

**Published:** 2023-10-11

**Authors:** Younghyun Kim, Jaewoong Kim, Hyeongsam Park, Sungbin Hong, Changmin Pyo, Gyuhae Park

**Affiliations:** 1 Automotive Materials & Components R&D Group, Korea Institute of Industrial Technology, Gwangju 61012, Republic of Korea; kyh1927@kitech.re.kr (Y.K.); kjw0607@kitech.re.kr (J.K.); 2 School of Mechanical Engineering, Chonnam National University, Gwangju 61186, Republic of Korea; 3 Department of Computer Engineering, Chonnam National University, Gwangju 61186, Republic of Korea; 157485@jnu.ac.kr; 4 Naval Architecture and Ocean Engineering, Seoul National University, Seoul 08826, Republic of Korea; njjo177@hshi.co.kr

**Keywords:** flux core arc welding, 9% nickel steel (ASTM A553-1), Goldak welding heat source model, Evolutionary optimization algorithm, simplification model

## Abstract

The International Maritime Organization (IMO) is tightening regulations on air pollutants. Consequently, more LNG-powered ships are being used to adhere to the sulfur oxide regulations. Among the tank materials for storing LNG, 9% nickel steel is widely used for cryogenic tanks and containers due to its high cryogenic impact toughness and high yield strength. Hence, numerous studies have sought to predict 9% nickel steel welding distortion. Previously, a methodology to derive the optimal parameters constituting the Goldak welding heat source for arc welding was developed. This was achieved by integrating heat transfer finite element analysis and optimization algorithms. However, this process is time-consuming, and the resulting shape of the weld differs by ~15% from its actual size. Therefore, this study proposes a simplified model to reduce the analysis time required for the arc welding process. Moreover, a new objective function and temperature constraints are presented to derive a more sophisticated heat source model for arc welding. As a result, the analysis time was reduced by ~70% compared to that previously reported, and the error rates of the weld geometry and HAZ size were within 10% and 15% of the actual weld, respectively. The findings of this study provide a strategy to rapidly predict welding distortion in the field, which can inform the revision of welding guidelines and overall welded structure designs.

## 1. Introduction

As the effects of climate change accelerate, air pollution is becoming a major environmental problem. Therefore, the International Maritime Organization (IMO) has continued to tighten regulations on air pollutants, such as sulfur oxides (SO_x_) and nitrogen oxides (NO_x_), to reduce air pollution emanating from ships. From 2020, marine fuel’s maximum permitted sulfur content has been reduced to 0.5% from 3.5% globally. Tier II regulations on nitrogen oxide (NO_x_) emissions have been in effect since 2011. Meanwhile, Tier III regulations, which apply only to Emission Control Areas (ECA), have been in effect since 2016. Consequently, ships operating in ECAs must be equipped with certified Tier III engines [1,2,3].

The shipping industry developed measures to adapt to these regulations, including using low-sulfur fuel, installing scrubbers, and operating LNG-powered ships [4,5]. Among these measures, LNG-fueled ships are gaining increased attention from shipowners as they significantly reduce air pollutant emissions, including SO_x_ and NO_x_. They are also more cost-effective than ships that use conventional fuels, which is advantageous regarding overall operating costs [6,7]. Compared to heavy fuel oils, liquefied natural gas (LNG) reduces the emission of NO_x_ by 85–95%, CO_2_ by 20%, and SO_x_ by ~100%, enabling shipowners to comply with IMO environmental regulations [8,9]. When extracted, LNG fuel exists as natural gas and occupies a large volume. However, when cooled to temperatures below −163 °C (−261 °F), LNG undergoes liquefaction, and its volume is reduced to approximately 1/600th of its gaseous state [10,11,12,13,14]. Therefore, it is essential to develop tanks suitable for storing LNG fuel. 

The IGC code specifies the materials that are suitable for use in storage tanks, including 304L stainless steel, 316L stainless steel, aluminum 5038-O, high manganese steel, 36% nickel steel, and 9% nickel steel, which have cryogenic toughness and a low thermal expansion coefficient. Among them, 9% nickel steel is widely used for producing the cryogenic equipment and containers of LNG ships [15]. However, certain issues persist, including high-temperature-induced cracking of the weld metal and heat-affected zone, and magnetism-induced arc blow during welding, a crucial process in cryogenic equipment and container production [16]. Hence, many studies have sought to improve the 9% nickel steel welding efficiency. For instance, Kim et al. performed butt welding via shielded metal arc welding (SMAW), submerged arc welding (SAW), and flux-cored arc welding (FCAW) on 9% nickel steel. They then performed impact testing at cryogenic temperature (−196 °C) and compared the results [16]. Meanwhile, Pyo conducted BOP tests on four shielding gases using fiber laser welding with 9% nickel steel and compared the penetration and HAZ depth through cross-sectional analysis [17]. Park et al. performed butt welding with super-TIG using Alloy625 filler metal and FCAW using Alloy600 filler metal on 9% nickel steel. They then compared the welds by conducting mechanical tests [18]. Xu et al. applied deep penetration keyhole tungsten inert gas (K-TIG) welding on 9% nickel steel. They analyzed the relationship between the grain size of martensite and the mechanical properties of the weld via cryogenic (−196 °C) impact tests and microstructure investigation [19]. Gook et al. performed analyses on Ytterbium fiber laser welding after preheating 9% nickel steel. Through cryogenic (−196 °C) impact testing, they established the optimal preheating temperature range to produce a sufficiently high-impact toughness comparable to the base metal [20].

FCAW has exhibited high efficiency as it has been used to develop welded wires with toughness and strength equivalent to the base metal. In turn, FCAW research is underway on cryogenic materials. For instance, Kim performed FCAW using hot steroid series DW-709SP filler metal on 9% nickel steel and achieved weld reliability through tensile, bending, hardness, and cryogenic (−193 °C) impact testing, as required for WPS approval [21]. Meanwhile, Mu et al. used DW-N625 filler metal on 9% nickel steel and performed crack tip opening displacement (CTOD) testing at room temperature (23 °C) and cryogenic temperature (−193 °C); they also observed the microstructure microscopically. Precipitation from TCP and carbides was observed on the weld, confirming that the number of precipitates depending on the welding conditions affected the fracture toughness. They also found that crack propagation caused by precipitation often occurs at cryogenic temperatures [22]. Additionally, Park et al. measured the bead geometry, area, and hardness of welds created via FCAW on 9% nickel steel. They found that the hardness varies depending on the ratio of wires mixed in the weld. Moreover, the weld quality decreases when the mixing ratio is ≥15.0%; the optimal welding conditions were derived using the multi-objective optimization (MOO) algorithm [23]. Moshtaghi et al. studied the FCAW and SMAW welding behavior of Ni addition ferritic weldments, confirming that applying FCAW increases the density of high-angle grain boundaries compared to SMAW [24].

Welding involves melting a localized area of material. The weld zone rapidly reaches a high temperature and then rapidly cools through heat transfer to a low-temperature area with a large volume. This drastic temperature change alters the material’s mechanical properties and causes residual stress and distortion of the welding, resulting in increased cost and time spent due to the need to select the proper welding conditions and equipment. In addition, the strength and dimensional accuracy of the welded structure is reduced. Therefore, many analytical studies have been conducted to reduce the required trial and error through finite element analysis. For example, Kim et al. conducted a 2D finite element analysis to investigate the residual stress and temperature distribution while welding 9% nickel steel plates. The finite element analysis results were consistent with the actual weld geometry and temperature distribution; the resulting residual stress was most apparent in the longitudinal direction of the weld line [25]. Manurung et al. performed multipass GMAW on a T-shaped structure comprising low manganese carbon steel and compared the weld temperature distribution and thermal distortion with 3D finite element analysis [26]. 

Meanwhile, Deng et al. performed CO_2_ welding on a thin panel structure made of 570 MPa thermo-mechanical control press (TMCP) steel. They then developed a welding distortion prediction method by comparing and analyzing the effects of thermal distortion, welding heat input, and sequence through 3D finite element analysis [27]. García-García et al. performed butt welding of 5.8 mm twinning-induced plasticity (TWIP) steel plates via the GTAW process. The temperature distribution according to the heat input was confirmed through 3D finite element analysis and was compared with the microstructure results of the welded specimens [28]. Moreover, Pyo et al. estimated the parameters of the arc heat source based on the results of applying FCAW to 9% nickel steel [29]. Collectively, this research was performed to establish a methodology to derive the main parameters constituting the welding heat source for each welding condition using bead-on-plate experiments, heat transfer finite element analysis, and optimization algorithms. However, the global optimization algorithm had to perform the time-consuming process of comparing 2000 candidates, while the size of the actual fusion zone and the analysis result differed by ~14%. This difference was caused by controlling only the heat-affected zone (HAZ) size through the objective function and not the fusion zone size.

Therefore, this study proposes a simplified model to address the slow analysis speed and presents precise objective functions and constraints to derive an arc welding heat source model comprising the fusion zone and HAZ sizes. Based on this model, the main parameters of the heat source were derived for three welding conditions, including those from the previous study. The derived heat source and weld geometry were determined experimentally and subsequently compared and analyzed; the differences between the simplified analysis model and the 3D-based full model were also compared. The simplified analysis model reduced analysis time by >70% compared to the previous study. Moreover, the error rates of the weld geometry and HAZ size compared to the actual weld were within 10% and 15%, respectively. Deriving an arc welding heat source model with a sophisticated fusion zone facilitated the prediction of welding distortion that occurs during arc welding with a small error rate. Hence, the findings of this study may help establish guidelines for welding in the field with 9% nickel steel and evaluate the changes in the welding location and structure design in consideration of the amount of distortion caused by thermal contraction or expansion. Given that this method uses analytics to predict welding distortion, the associated cost will be reduced as it will facilitate troubleshooting within the design stage, avoiding the requirement for actual experiments.

## 2. Welding Experiments and Results

### 2.1. Welding Materials and Conditions

**Figure 1:**
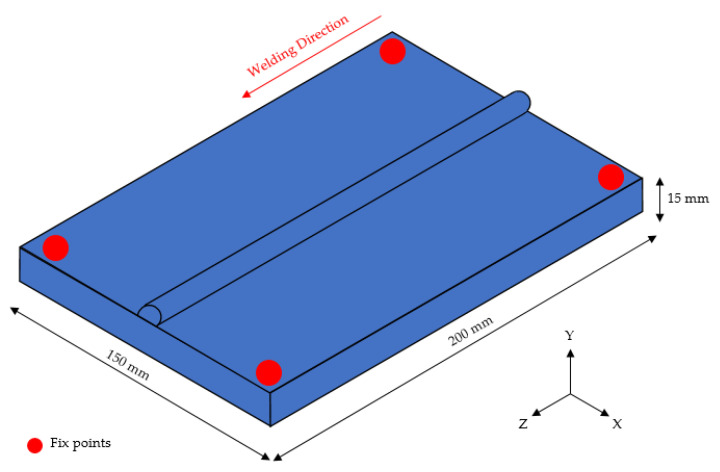
Schematic diagram of welding.

This study used 9% nickel steel (ASTM A553-1) [30], and bead-on-plate (BOP) welding was performed using the FCAW process. The dimensions of the test specimen plates were 150 mm (W) × 200 mm (L) and were 15 mm thick. Welding was performed by fixing the ends of the four corners. Figure 1 shows a schematic diagram, including the welding direction. The wire used in the experiment was AWS A5.14 ERNiMo-8 (KOBELCO, TG-S709S, Changwon-si, Korea) with a diameter of 1.2 mm. Table 1 shows the chemical composition of ASTM A553-1 [30], and Table 2 presents its mechanical properties [30].

Welding conditions included current, voltage, and welding speed, the main variables that affect heat input. The voltage was fixed at 15 V and the welding speed at 0.4 m/min; the experiment was conducted by changing only the current. Table 3 shows the welding conditions, including the shielding gas.

### 2.2. Cross-Section Analysis Results

**Figure 2:**
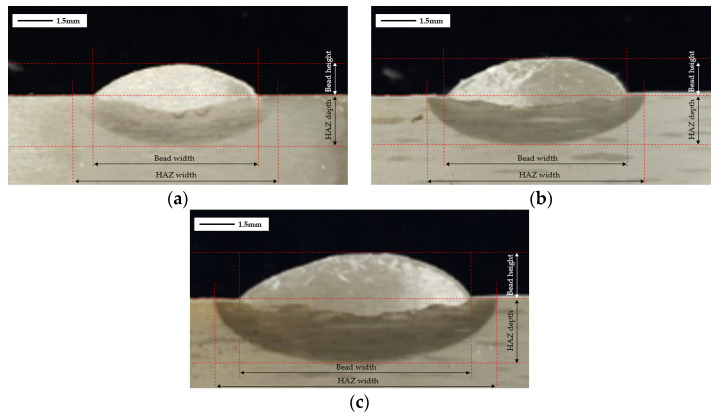
(**a**) Case 1, (**b**) Case 2, (**c**) Case 3, Parameter measurement location for each.

**Figure 3:**
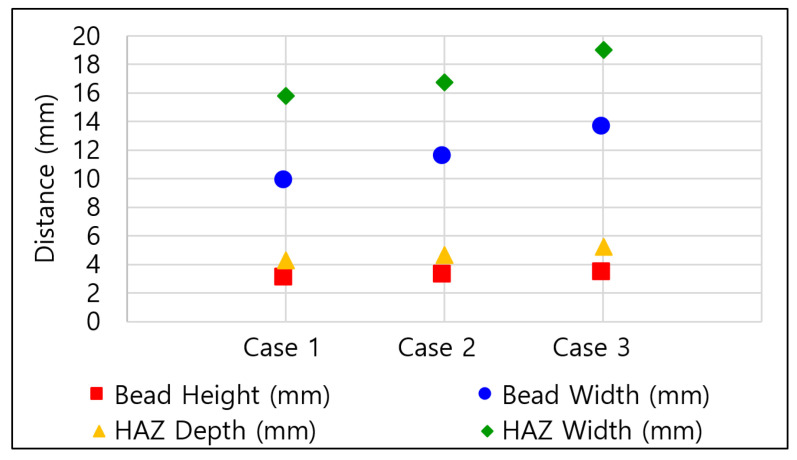
Cross-sectional analysis results by case.

FCAW welding was performed with a 600 A class welding machine (ProPAC, HYOSUNG, Mapo-gu, Seoul, Republic of Korea) comprising a torch, weld feeder, direct welding carriage, and rail. After BOP welding with the FCAW equipment, the specimens were cut transversely at the center point of the weld line to observe the weld geometry. The cut sections were sprayed with 90% ethanol and 10% nitric acid, and the shape of the weld was analyzed using an optical microscope (EGVM 35B, EG Tech, Anyang, Republic of Korea) to measure the main welding parameters (bead width, bead height, HAZ width, and HAZ height). Figure 2 shows the parameter measurement locations for each case, and Table 4 and Figure 3 shows the measurements. As a result of cross-sectional analysis, it was confirmed that the measurement parameters increased every time the current value increased by 10A. Bead Height increased by ~6.51%, bead width by ~18.00%, HAZ depth by ~12.39%, and HAZ width by ~9.73%. The size of the fusion zone and HAZ area increased as the magnitude of the current increased. The difference might be due to the increase in heat input.

## 3. Deriving the Heat Source Model

### 3.1. Process for Deriving the Heat Source

The process comprised BOP welding, measuring the weld geometry, and deriving the heat source results. Abaqus (Ver. 2020, Dassault Systems Simulia Corp, Johnston, RI, USA) was used as the finite element analysis program for heat transfer analysis. Considering the previously reported high reliability of Fortran user subroutines [31,32,33,34,35], they were used to implement the moving heat source (Fortan Ver. 17.0, Intel, San Jose, CA, USA).

**Figure 4:**
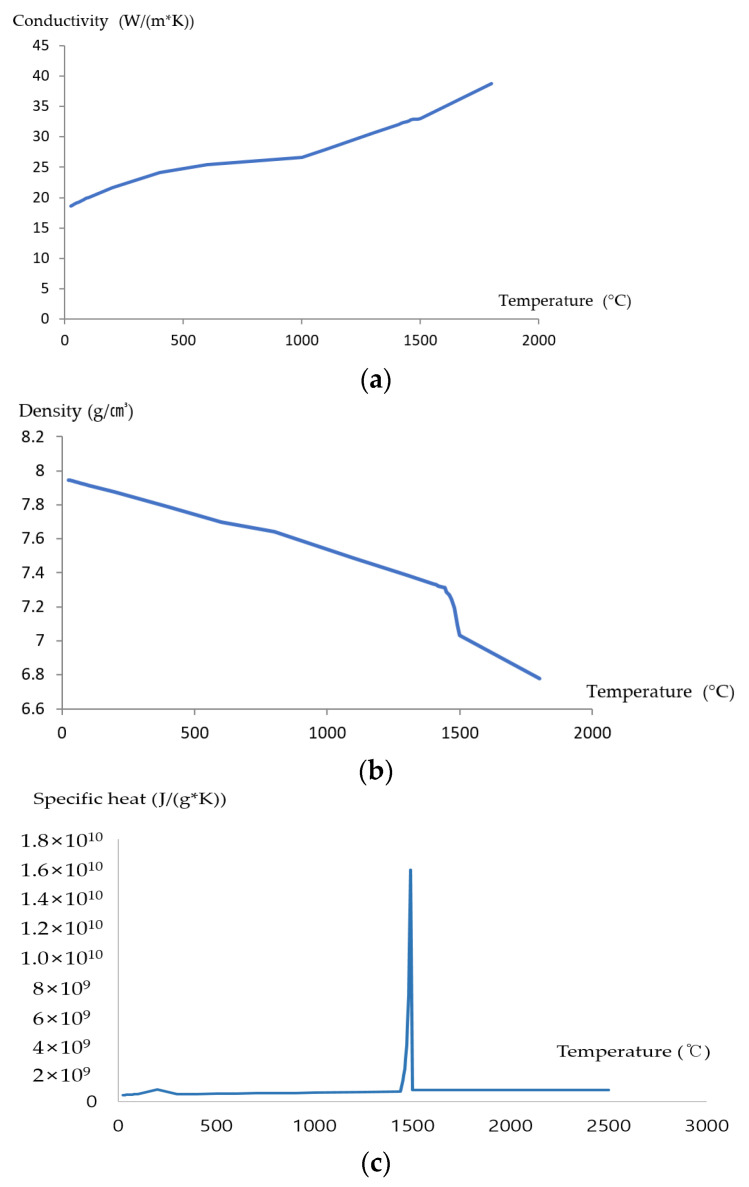
Material properties of 9% nickel steel by temperature (**a**) Conductivity, (**b**) Density, (**c**) Specific heat.

Welding heat transfer analysis requires temperature-specific mechanical properties of the material, especially data at high temperatures. As the Jmatpro program has been used to derive the mechanical properties of metals [36,37], it was employed in this study to derive temperature-specific physical properties of 9% nickel steel. In addition, heat transfer analysis was performed by applying physical property data according to temperature to the analysis model. Figure 4 shows the thermal conductivity, specific heat, and density by temperature (25–2500 °C). A sharp decrease in density values was observed around the melting temperature of approximately 1500 °C for 9% nickel steel.

**Figure 5:**
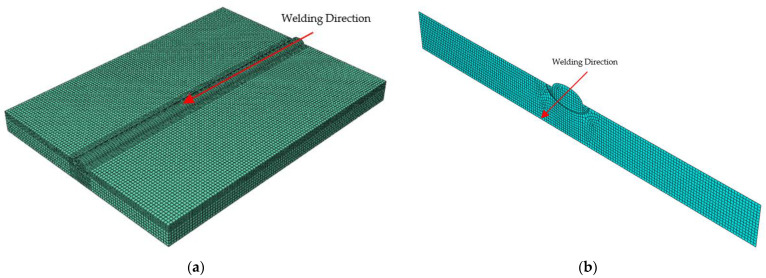
Original dimension model and simplified model (**a**) Original dimension model (with coarse meshes), (**b**) Simplified model (with fine meshes).

A finite element analysis model was constructed based on the experiments, and a moving heat source was applied to the center of the 150 mm wide, 200 mm long, and 15 mm thick sample to analyze the welding process. For 3D analysis, the mesh size was set to 2 mm, and ~90,000 meshes were used. Its accuracy was lower than a 2D model with a mesh size of 0.8 mm. Hence, as more than 2000 comparative analyses are required in this study, the 2D shell model was simplified to shorten the analysis time (Figure 5). The simplified model had a DC2D4 type mesh with a 4-node linear heat transfer quadrilateral and ~7000 meshes. The analysis quality was validated using a previously reported method [38]. The optimal arc heat source derived by the algorithm was applied to the 3D and 2D models to perform heat transfer analysis and compare the results. The convective heat transfer coefficient was set to 10 W/m^2^K, the emissivity to 0.8, and the air temperature to 20 °C [29].

### 3.2. Simplifying the Heat Transfer Analysis Model

**Figure 6:**
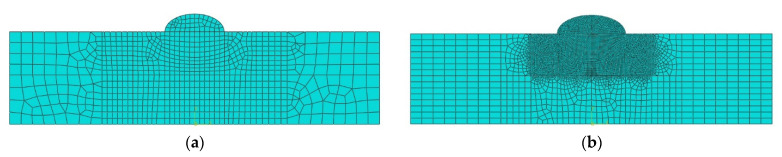
Mesh shape by zone (**a**) Simplification model, (**b**) The previous study model.

Due to the difficulty associated with conducting experiments in the field, welding heat transfer analysis is often used to identify trends. However, the heat transfer analysis performed in previous studies are time intensive due to the large number of meshes, impeding the ability to rapidly identify trends in the field. Our previous study used a model with a 0.15 mm mesh and ~9000 meshes. However, in this study, the mesh size was reduced to 0.80 mm, and the count was increased to ~1200 to increase the analysis speed. Moreover, the model was simplified by ~80% compared to the previous study to compare and analyze the temperature distribution of the weld. As a result, the error rate of the weld geometry was ~20%, and the error rate of the HAZ size was ~7% compared to the previous experimental values. Meanwhile, in this study, the weld geometry and the HAZ size error rates were within 10% and 15% of the experimental values, respectively. Moreover, the analysis time decreased by more than 70%. More specifically, the previous model required 1680 s to analyze one model while the simplified model required 420 s. Therefore, applying a simplified model was considered more appropriate to identify trends and was used in the current study to derive the heat source. Figure 6 compares the simplified and previous models.

### 3.3. Goldak Model Heat Source

**Figure 7:**
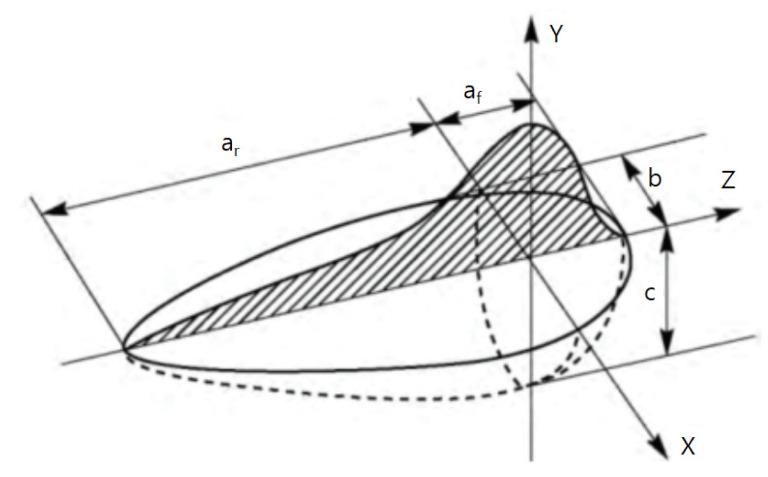
Heat distribution of Goldak model.

The Goldak double ellipsoid model (Figure 7) is the most used heat source model in finite element analysis of arc welding, defined by the governing Equations (1)–(5). In Equations (2) and (3), the parameters a_f_, a_r_, b, and c are independent so that they can have different values for the front and back heat sources. Appropriate parameter values are required to simulate a geometry similar to the actual heat source; hence, many studies have sought to estimate these values. Farias et al. proposed a new heat source geometry based on Goldak’s double ellipsoid model and applied a genetic algorithm to estimate the heat source for arc welding [39,40]. Meanwhile, Pyo used adaptive simulated annealing (ASA) to estimate the arc welding heat source [41].

Q = μVI(1)

(2) $$q_{f}\left(x,y,z,t\right)=\frac{6\sqrt{3}f_{f}Q}{a_{f}\mathrm{bc}\pi }\exp\left(-3\left(\frac{{\left(z-\mathrm{vt}-z_{0}\right)}^{2}}{a_{f}{}^{2}}+\frac{y^{2}}{c^{2}}+\frac{x^{2}}{b}\right)\right)$$

(3) $$q_{r}\left(x,y,z,t\right)=\frac{6\sqrt{3}f_{r}Q}{a_{r}\mathrm{bc}\pi }\exp\left(-3\left(\frac{{\left(z-\mathrm{vt}-z_{0}\right)}^{2}}{a_{r}{}^{2}}+\frac{y^{2}}{c^{2}}+\frac{x^{2}}{b^{2}}\right)\right)$$

(4) $$f_{f}=\frac{2a_{r}}{a_{f}+a_{r}}$$

(5) $$f_{r}=\frac{2a_{f}}{a_{f}+a_{r}}$$

Q: Effective heat energy

μ: Welding efficiency

V: Voltage

I: Current

$f_{f}$: Fraction of heat deposited at the front ellipsoid

$f_{r}$: Fraction of heat deposited at the rear ellipsoid

v: Velocity of heat source

a_f_, a_r_, b, c: Dimension parameters (Shown in Figure 7)

## 4. Optimization Algorithm

### 4.1. Software

The optimization algorithm program used in this study was Isight (Ver. 2020, Dassault Systems Simulia Corp, Johnston, RI, USA), designed by the same company that created Abaqus. Many studies have been conducted to design optimization algorithms using Isight [42,43,44,45].

### 4.2. Algorithm Process

**Figure 8:**
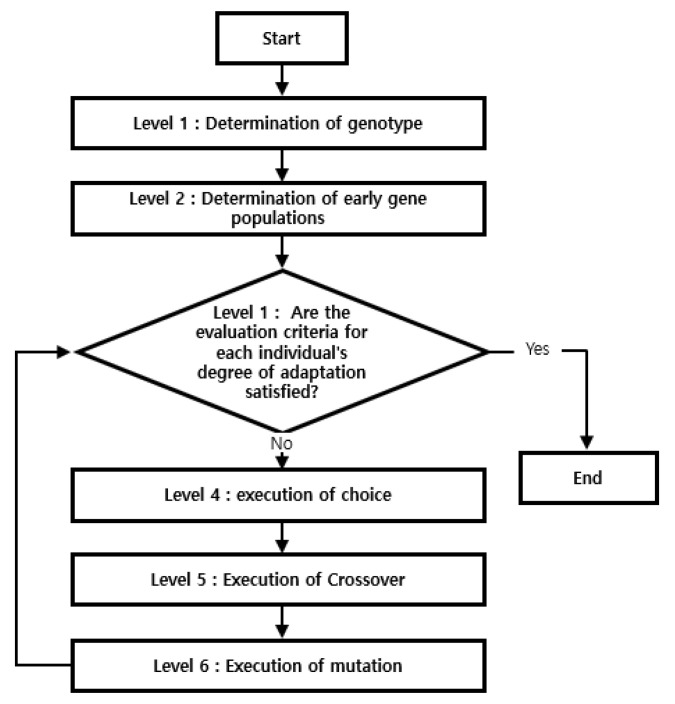
Evolutionary optimization algorithm [49].

Heat transfer analysis was repeated by altering six parameters, including the welding efficiency and the Goldak model parameters. It was, therefore, necessary to determine the parameter value with the closest temperature distribution based on comparing the analysis results with the actual HAZ size in Table 4. To find the optimal parameters of the arc heat source, the evolutionary optimization algorithm (Evol) method was applied (Figure 8) [46]. Evol is an evolutionary algorithm-based method that randomly adds normally distributed values to each design variable and applies mutation (standard deviation of the normal distribution) to identify the optimal value. The parental generation is randomly selected, and mutation operations are used to generate offspring. After evaluating the results produced by the mutations, the most suitable results replace the parents of the next generation; this process is repeated to converge with the objective function. In this way, Evol can solve problems with nonlinear constraints as it does not require any information other than that derived from the object evaluation method. As such, it has been used in many optimization processes [47,48].

### 4.3. Limiting Parameter Temperature Range

**Figure 9:**
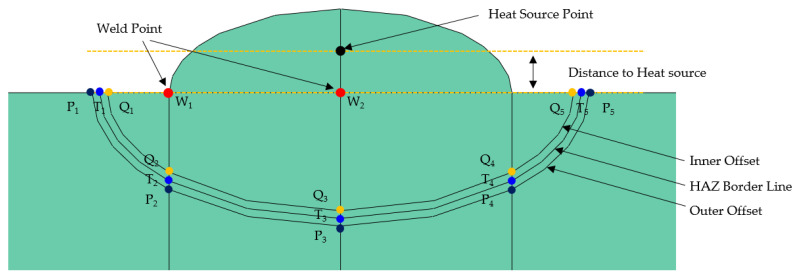
Offset shape, temperature checking position, definition of distance to heat source.

Based on the test results in Table 4, a 1.0 mm offset between the weld bead and HAZ boundary and inside and outside the HAZ border was applied to the model. In the previous study, temperatures were checked at five points at the offsets inside (Q_1–5_) and outside (P_1–5_) the HAZ; however, the temperature at the fusion zone was not recorded. Consequently, it was impossible to control or accurately predict the size of the fusion zone. Therefore, in this study’s analysis model, the temperatures were checked at two weld points (W_1–2_), five points at the offsets inside (Q_1–5_) and outside (P_1–5_) the HAZ, and five points at the HAZ boundary (T_1–5_) (Figure 9). The weld points were at locations completely melted in the cross-sectional analysis (Figure 2), the end of the bead, and where the centerline of the bead met the base metal. Adding weld points reduces the range in finding an appropriate solution for the multivariate function and helps derive an arc welding heat source with a precise weld.

The following temperature constraints were assigned to derive a highly consistent heat source through the optimization algorithm: the weld point must exceed 1450 °C and the inner offset point must reach 600 °C at least once, while the outer offset point cannot exceed 600 °C. The temperatures of both the inner and outer offset constraints were set to 600 °C as carbon steel has an Ac1 point where its structure changes at temperatures above 600 °C. Therefore, 600 °C was selected as the temperature that separates the HAZ, and 1450 °C was applied for the fusion zone as melting occurs above this temperature. In the previous study, the sum of the differences between the maximum values of the inner and outer points was set as the objective function. However, as the offset interval widens, the range of the objective function value widens and becomes less consistent. Therefore, in this study, the HAZ boundary point was included in the objective function, and Equation (6) was applied by setting the value obtained by subtracting 600 °C from the sum of the maximum values as the objective function, as follows:

(6) $$\mathrm{Objective}\;\mathrm{funtion}=\;\mathrm{Min}\overset{k}{\underset{n=1}{\sum }}\left|T_{\mathrm{Tn}}-h\right|$$

T_Tn_: Temperature of HAZ Border Line check point n

n: Number of check point (n = 1, 2, 3, 4, 5)

k: Total number of check points (k = 5)

h: HAZ boundary temperature

### 4.4. Setting Variables and Ranges

This study sought to identify the optimal values of the parameters that minimize the objective function while satisfying the temperature constraints. There were six parameters in total. The parameters of the Goldak model, welding efficiency, and distance from the heat source were set as variables. As shown in Table 5, the upper bound was increased to find an optimal solution within a wide range. The welding efficiency was set to 5% (±2.5%) with 0.8 as the reference value. The variables a_f_, a_r_/a_f_, b, and c were set to a relatively wide range. The distance to the heat source range was within the bead stacked on the upper part of the base metal, and Table 6 shows the values set as the bead’s height for each case in Table 4. Additionally, Figure 9 presents the distance to heat source locations and ranges.

## 5. Results and Analysis

### 5.1. Deriving the Heat Source Parameters and Heat Transfer Analysis Results

Approximately 2000 candidates were compared using Isight. The parameter with the smallest objective function was derived and selected as the optimal value. Table 7 shows the derived parameter values, all were within the bound range established in Table 5. In addition, Table 7 shows a comparison between parameters from previous studies.

The welding heat input differed for each case (Table 3). The a_r_ and c parameters, constituting the heat source, showed a tendency, while a_f_ and b did not. This might have been caused by the offset range of 1.0 mm. Hence, the correlation between the heat input amount and the heat source parameter cannot be shown.

Navid et al. derived the optimal parameters of the Goldak heat source using the artificial neural network (ANN) method and compared the actual experimental results with the simulation results. They confirmed that the cross-section of the actual welding and simulation results were similar. However, the optimal parameters of the Goldak heat source derived from the ANN were also difficult to correlate with welding conditions [50].

Table 8 shows the values at the temperature constraint points obtained from the heat transfer analysis. Under all conditions, the maximum temperature at the outer offset P_1-3_ points did not exceed 600 °C, while those at all of the inner offset Q_1-3_ points were above 600 °C. The maximum temperature at the weld point was >1450 °C, and the temperature was ~600 °C at the HAZ boundary.

### 5.2. Heat Transfer Analysis Results by Welding Conditions

**Figure 10:**
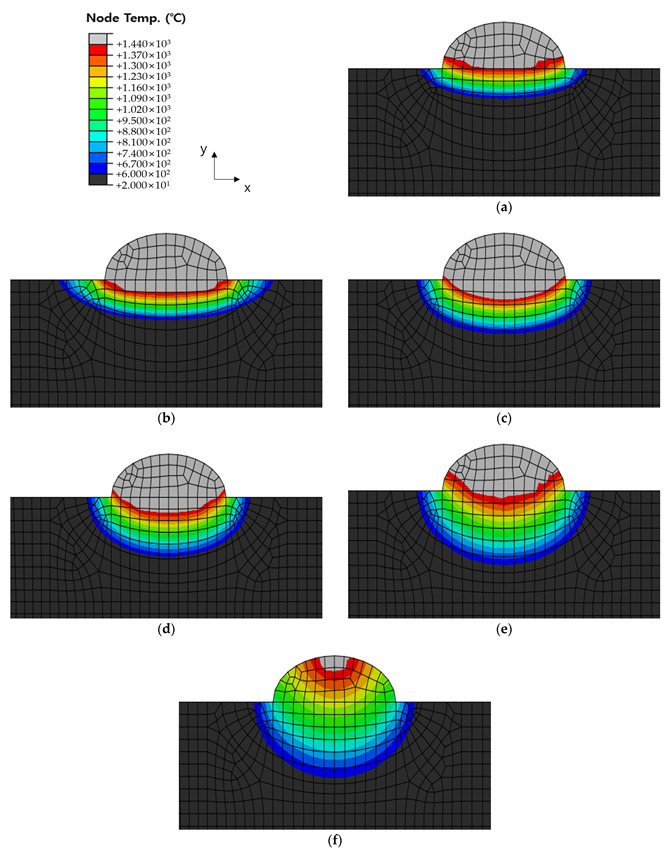
Temperature distribution by timeline before and after welding of Case 1. Times were (**a**) −0.1 s (before the heat source arrives); (**b**) 0 s (when the heat source is directly above); (**c**) 0.5 s elapsed; (**d**) 1.0 s elapsed; (**e**) 2.0 s elapsed; (**f**) 4.0 s elapsed.

**Figure 11:**
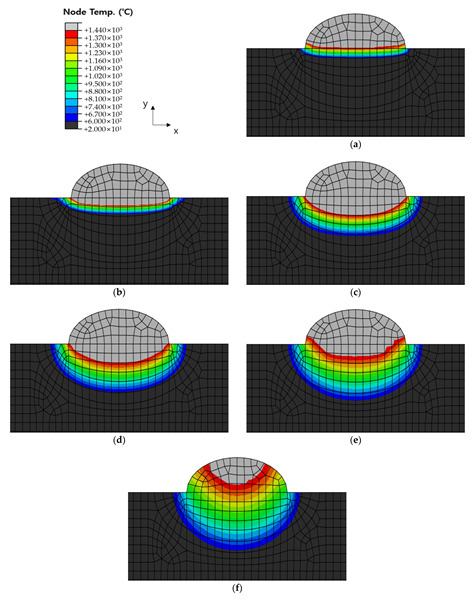
Temperature distribution by timeline before and after welding of Case 2. Times were (**a**) −0.1 s (before the heat source arrives); (**b**) 0 s (when the heat source is directly above); (**c**) 0.5 s elapsed; (**d**) 1.0 s elapsed; (**e**) 2.0 s elapsed; (**f**) 4.0 s elapsed.

**Figure 12:**
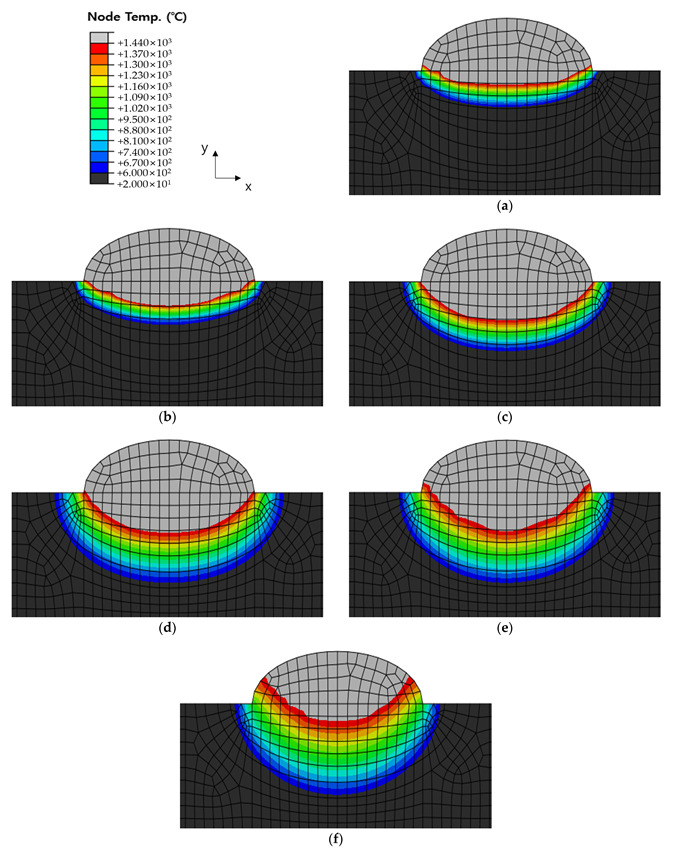
Temperature distribution by timeline before and after welding of Case 3. Times were (**a**) −0.1 s (before the heat source arrives); (**b**) 0 s (when the heat source is directly above); (**c**) 0.5 s elapsed; (**d**) 1.0 s elapsed; (**e**) 2.0 s elapsed; (**f**) 4.0 s elapsed.

Figure 10, Figure 11 and Figure 12 show the results of analyzing heat transfer in each case as time elapsed. The results were then compared from 0.1 s before the heat source arrived to 4.0 s after the heat source passed. In addition, the maximum HAZ width and HAZ depth were measured to compare the experimental and analytical results and obtain the error rate. The maximum HAZ width of Cases 1 and 2 was reached the instant the heat source arrived, and the maximum HAZ depth was reached 4.0 s after the heat source passed. The maximum HAZ width in Case 3 also was reached 1.0 s after the heat source arrived, and the maximum HAZ depth was reached 4.0 s after the heat source passed.

The maximum HAZ width and depth were measured from the heat transfer analysis results applying the simplified model for each case, and compared with the actual experimental results in Table 4 (Table 9).

Case 1 had the smallest HAZ width error rate (1.96%), however, all cases had error rates within 4.81% and HAZ depth error rates within 14.73%. Hence, the 1.0 mm offset range significantly affects the HAZ depth measurement, which is relatively small compared to the HAZ width measurement. To find a more precise heat source model, reducing the offset range may reduce the error rate.

### 5.3. Comparing the Heat Transfer Results between the Original Dimension Model and the Simplified Model

**Figure 13:**
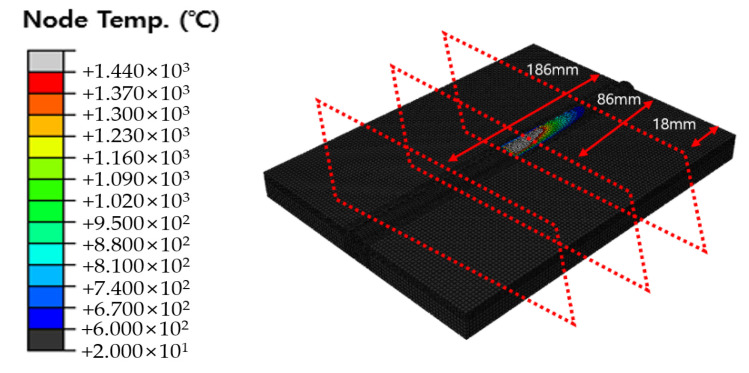
Section check position in the heat transfer analysis for the original dimension model.

**Figure 14:**
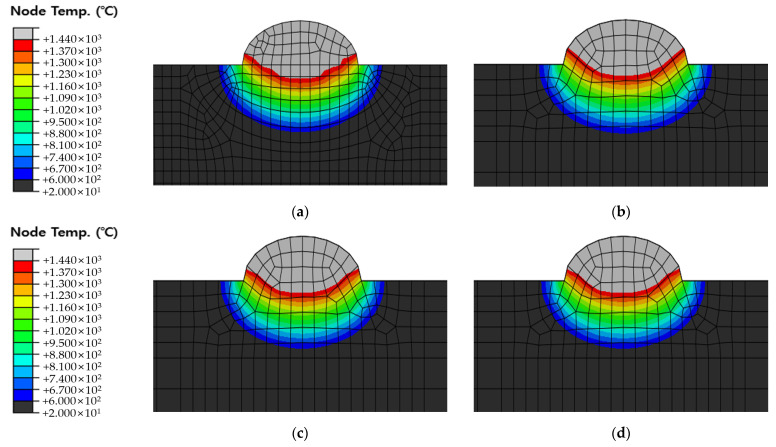
Comparison of temperature distribution of Case 1 and Original dimension model 1.6 s after the heat source has passed (**a**) Simplified model with fine meshes; (**b**) 18 mm position of original dimension model; (**c**) 86 mm position of original dimension model; (**d**) 186 mm position of original dimension model.

**Figure 15:**
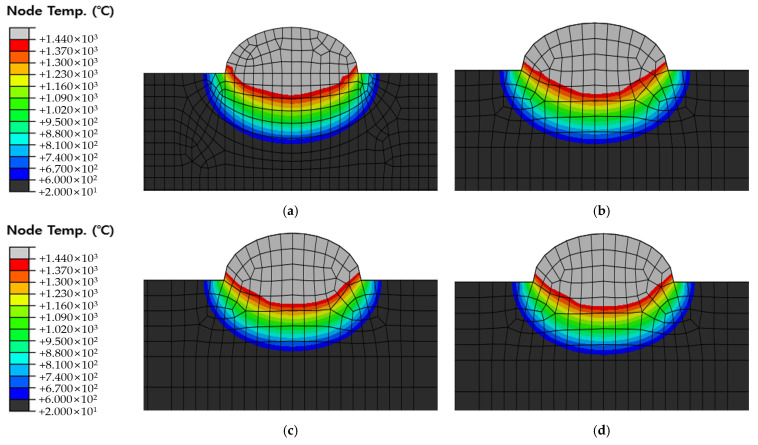
Comparison of temperature distribution of Case 2 and Original dimension model 1.6 s after the heat source has passed (**a**) Simplified model with fine meshes; (**b**) 18 mm position of original dimension model; (**c**) 86 mm position of original dimension model; (**d**) 186 mm position of original dimension model.

**Figure 16:**
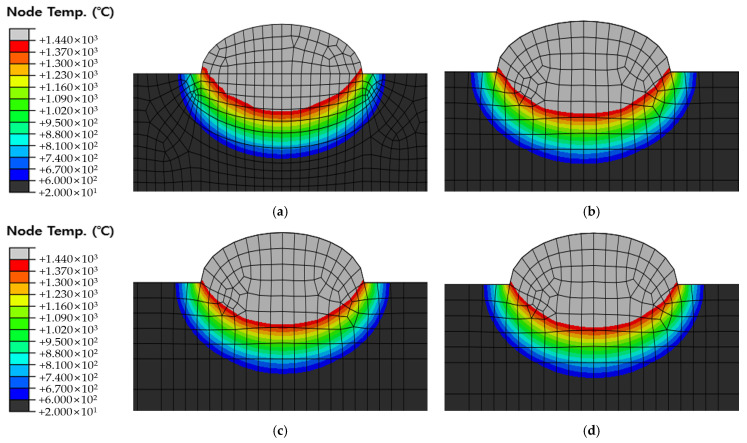
Comparison of temperature distribution of Case 3 and Original dimension model 1.6 s after the heat source has passed (**a**) Simplified model with fine meshes; (**b**) 18 mm position of original dimension model; (**c**) 86 mm position of original dimension model; (**d**) 186 mm position of original dimension model.

The original dimension model was simplified, as shown in Figure 5 and Figure 6, and the optimal heat source parameter values were derived by applying the optimization algorithm and objective function to the simplified model. Therefore, it is necessary to compare and verify the original dimension model and the simplified model. The default mesh size of the original dimension model was set to 2 mm and 1 mm within 10 mm on both sides from the center of the weld. The DC3D8R element was used as the mesh type; hence, an 8-node linear heat transfer brick and reduced integration were applied. The mesh count for Cases 1, 2, and 3 were 90,300, 90,900, and 91,900, respectively. The temperature distribution was checked 1.6 s after the heat source passed at 18 mm, 86 mm, and 186 mm of the original dimension model and compared with the simplified model. Figure 13 shows the positions for checking the temperature distribution. Figure 14, Figure 15 and Figure 16 show the comparison of Cases 1, 2, and 3 results, respectively (4-node linear heat transfer quadrilateral).

When visually comparing the temperature distribution of the simplified model and the original dimension model, it was confirmed that the sizes of the melted zone and HAZ area were similar until 1.6 s after the heat source passed. However, the measurement results for HAZ width and HAZ depth showed a slight difference. This is attributed to the original dimension model being a 3D model where heat transfer occurs in all directions.

Table 10 shows the HAZ width and HAZ depth compared to the simplified model when the heat source passes through the 18 mm, 86 mm, and 186 mm points of the original dimension model. In all cases, the size at 186 mm was significantly larger than at 18 mm. This is attributed to the heat generated by the heat source, indicating that heat transfer occurs effectively as the heat source moves. Subsequently, we compared the average measurements of the original dimension model based on the values from the simplified model as a reference. In Case 1, HAZ width and HAZ depth showed error rates of 11.01% and 12.75%, respectively. In Case 2, the error rates were 13.49% and 11.97%, and 12.63% and 9.53% in Case 3. All error rates were confirmed to be within 14%. Hence, it is considered advantageous to apply the simplified model in field scenarios where speed is crucial. Considering that the results from the simplified model are slightly higher than the actual experimental results (Table 9), we can conclude that the original dimension model provides heat transfer results with higher consistency. However, there is a need to develop a more sophisticated analysis model while ensuring high speed.

## 6. Conclusions

This sought to improve the slow interpretation speed, which hinders the procurement of arc welding heat sources, by applying optimization algorithms. More specifically, we aimed to improve the interpretation speed by devising simplified interpretation models. In addition, temperature constraints were added to compensate for the shortcomings of existing studies, and a new objective function was proposed to ensure consistency. The primary findings are summarized as follows:

(1) The heat transfer analysis results show that the optimal parameters of the Goldak model derived by the optimization algorithm satisfied all temperature constraints.
(2) The model applied in the previous study was simplified to speed up the analysis process, which increased the analysis speed by about 70%.
(3) A heat source model that melts the entire weld bead was derived through the temperature constraints of the weld point, and a consistent HAZ area was simulated through a new objective function.
(4) By comparing the HAZ width and HAZ depth of the simplified model with the actual weld cross-sections, the HAZ width and depth exhibited maximum differences of 4.81% and 14.73%, respectively.
(5) By comparing the heat transfer analysis results between the simplified model and the original dimension model, the HAZ width and depth showed maximum differences of 13.49% and 12.75%, respectively.
(6) This study applied a simplified model based on the HAZ size to rapidly derive optimal heat source parameters and identify the weld geometry through heat transfer analysis, which was considered to save time.

## Figures and Tables

**Table 1:** Chemical composition of base metal and filler wire (wt.%) [30].

	C	Si	Mn	S	P	Ni	Fe
Parent material	0.05	0.67	0.004	0.003	0.25	9.02	Bal.
Welding consumables	0.02	0.02	0.1	0.001	0.001	69.8	Bal.

**Table 2:** Mechanical properties of 9% nickel steel [30].

Yield Strength (MPa)	Tensile Strength (MPa)	Elongation (%)	Hardness (HV)
651.6	701.1	26.6	243

**Table 3:** FCAW parameters and experimental condition.

Case	Current (A)	Voltage (V)	Welding Speed (m/min)	Shielding Gas (L/min)
Case 1	150	25	0.4	18
Case 2	160	25	0.4	18
Case 3	170	25	0.4	18

**Table 4:** Key parameter values.

Case	Bead Height (mm)	Bead Width (mm)	HAZ Depth (mm)	HAZ Width (mm)
Case 1	2.90	9.68	4.14	15.74
Case 2	3.09	11.35	4.66	16.64
Case 3	3.29	13.48	5.23	18.92

**Table 5:** Variables and ranges.

Variable	Lower Bound	Upper Bound
μ (W/W)	0.78	0.82
a_f_ (mm)	1.0	15.0
a_r_/a_f_ (mm/mm)	1.5	7.0
b (mm)	1.0	20.0
c (mm)	1.0	15.0

**Table 6:** Distance to heat source ranges.

	L (Distance to Heat Source, mm)	
	Lower Bound	Upper Bound
Case 1	0	2.90
Case 2	0	3.09
Case 3	0	3.29

**Table 7:** Derived heat source parameters.

Variable	Value		
	Case 1	Case 2	Case 3
μ (W/W)	0.82	0.81	0.82
a_f_ (mm)	2.96	1.84	7.72
a_r_/a_f_ (mm)	5.79	6.56	7
b (mm)	13.92	19.62	11.26
c (mm)	4.64	1.84	1.00
L (mm)	2.09	3.09	3.29

**Table 8:** Maximum temperature at each checkpoint per welding condition.

Temperature (°C)	Value		
	Case 1	Case 2	Case 3
P_1_	546.83	522.86	266.03
P_2_	413.66	389.53	407.15
P_3_	419.67	391.85	458.52
Q_1_	877.38	684.56	643.07
Q_2_	787.20	830.43	821.43
Q_3_	876.75	909.68	994.34
M_1_	1450.84	1701.07	1474.87
M_2_	2117.31	2692.81	3302.30
T_1_	705.26	596.45	425.85
T_2_	586.05	586.16	592.86
T_3_	618.90	612.57	691.02

**Table 9:** HAZ dimension and comparison.

Value	HAZ Width			HAZ Depth		
	FEM (mm)	Experiment (mm)	Difference (%)	FEM (mm)	Experiment (mm)	Difference (%)
Case 1	16.05	15.74	1.96	4.75	4.14	14.73
Case 2	15.84	16.64	4.81	5.20	4.66	11.59
Case 3	18.50	18.92	2.22	5.77	5.23	10.33

**Table 10:** HAZ size comparison between the original dimension and simplified models.

Value		Variables				
		Simplified Model (mm)	Original Dimension Model (mm)			Difference (%)
			18 mm	86 mm	186 mm	
HAZ Width	Case 1	13.83	12.25	12.28	12.39	11.01
	Case 2	14.70	12.60	12.71	12.84	13.49
	Case 3	16.89	14.62	14.70	14.95	12.63
HAZ Depth	Case 1	4.42	3.80	3.87	3.90	12.75
	Case 2	4.68	4.09	4.11	4.16	11.97
	Case 3	5.70	5.05	5.18	5.24	9.53

## Data Availability

The data presented in this study are available on request from the corresponding author.
